# Well-being predictors of body composition and associated behavioral risk factors in midlife/older women participating in a meditative movement intervention: an exploratory analysis

**DOI:** 10.1017/cts.2023.621

**Published:** 2023-09-04

**Authors:** Dara L. James, Linda K. Larkey, Kimberley Goldsmith, Bronwynne Evans, Ann Sebren, Nanako A. Hawley

**Affiliations:** 1 Edson College of Nursing and Health Innovation, Arizona State University, Tempe, AZ, USA; 2 Department of Biostatistics & Informatics, Inst. Of Psychiatry, King’s College London, London, UK; 3 College of Health Solutions, Arizona State University, Tempe, AZ, USA; 4 College of Arts and Sciences, University of South Alabama, Mobile, AL, USA

**Keywords:** Stress, sleep, eating, Tai Chi, Qigong

## Abstract

**Introduction:**

Greater than 40% of women are obese, a key risk factor for cardiometabolic, neurocognitive disease, mood disorders, and certain cancers. Obesity and unfavorable body composition can compromise physical and psychological health and well-being. Preliminary evidence demonstrates Meditative Movement (i.e., Tai Chi Easy) improves health outcomes and body composition among midlife/older women. This single-group pilot study explored relationships between well-being predictors related to body composition and associated behavioral risk factors in midlife/older women pre-to-post Tai Chi Easy intervention.

**Methods:**

Eligible women 45–75 years old, participated in once-weekly 30-minute Tai Chi Easy classes over 8-weeks. Pre/post-intervention data included self-report surveys and on-site body composition. Multivariate linear regression models were fitted with putative predictor variables having correlations *p*-values of 0.20 or less with sleep quality and eating behaviors.

**Results:**

Participants (*N* = 36) (*M* age = 53.7) were White (80.4%) and attended ≥ 4 years of college (70.6%). Analyses resulted in one independent variable per model as a predictor of the dependent variables of sleep quality and emotional eating. Results indicated: (1) stress explained 13.4% sleep quality variance (*F* (2, 20) = 2.71, *p* = 0.09) and (2) self-compassion explained 42.1% emotional eating variance (*F* (2, 31) = 12.54, *p* < .01).

**Conclusion:**

Findings suggest stress and self-compassion partially explain variance in the dependent variables of sleep quality and emotional eating, both associated behavioral risk factors of body composition. Additional research may guide interventions to test efficacy and examine mediators to improve well-being predictors, body composition, and associated behavioral risk factors among midlife/older women.

## Introduction

Well-being is a multifaceted construct relating to a general positive quality of life experience [1,2]. Well-being integrates physical, psychological, and emotional aspects of functioning, supporting a holistic approach to health interventions, and promotion [1,2]. Midlife and older women (i.e., into and beyond menopause) are at an elevated risk for health-related issues that negatively impact well-being [3–6]. Stress, weight gain, and adverse changes in body composition (e.g., increased fat mass and central adiposity, decreased fat-free mass) may contribute to the development of obesity and deleterious health outcomes among midlife/older women [4–7]. Although chronologically and biologically distinct, both stages in a woman’s life: (1) midlife (peri-menopause and menopause), and (2) older (post-menopause), are considered to be high-risk periods for weight gain and unfavorable changes in body composition posing a threat to physical and psychological health and well-being [4–7]. While body composition is comprised of various components, the ratio of fat mass to fat-free mass, in particular, is a well-known indicator of cardiometabolic risk factors (e.g., type 2 diabetes, hypertension) and is significantly associated with mortality [8–10]. Efforts to improve physical well-being through improved body composition are most frequently targeted via modifiable lifestyle interventions including physical activity and/or diet [11,12]; however, sustained results are limited and further, a myriad of psychological, behavioral, and physiological factors contribute to the complex etiology of obesity and subsequent outcomes [8–10]. Emerging as central to this compilation of behaviors that may impact obesity are poor sleep and emotional eating in response to stress. Research repeatedly demonstrates that poor sleep (e.g., quality, duration) is strongly associated with weight gain, compromised body composition, and obesity [13,14]. The restorative properties and process of sleep, specifically sleep quality, facilitate the regulation of the endocrine system (e.g., cortisol, leptin, ghrelin) and glucose metabolic function (e.g., insulin sensitivity), both of which play a significant role in weight management and body composition [15,16]. Additionally, poor sleep quality contributes to maladaptive weight-related behaviors including increased caloric consumption (i.e., high-fat/high-calorie foods), decreased physical activity, and increased sedentary time [15,16]. Sleep quality is paramount to healthy and/or improved body composition in addition to physical and psychological well-being.

Emotional eating, or the adverse behavior of eating in response to negative emotions [17], is strongly associated with weight gain and adverse changes in body composition [18,19]. Emotional eating may be triggered by a multitude of negative, or undesirable/uncomfortable emotions (e.g., stress, sadness, anxiety, depression, loneliness) [17], and further, in the absence of emotional regulation, the behavior of emotional eating may become a habitual pattern therefore contributing to weight gain, unfavorable body composition and obesity.

Quality of sleep has been attributed to emotional factors of well-being, which may contribute to poor sleep quality and/or increased emotional eating, leading to shifts in weight gain and body composition. Sleep is associated with mood states and varied emotions and is considered a bidirectional relationship such that improved sleep may contribute to improved mood/emotion and vice versa [20]. Further, improved sleep quality is associated with psychological well-being and the state and experience of being self-compassionate (i.e., being kind to oneself, experiencing feelings of caring and kindness toward oneself) such that higher self-compassion may help buffer the effects of stress, and therefore improve sleep quality [21].

A rapidly growing field of evidence supports the potential for mind-body interventions to improve various factors of well-being associated with obesity (e.g., stress, anxiety, sleep, emotional eating) [22–24], including weight loss and body composition outcomes [25–27]. One such modality of a mind-body intervention is Meditative Movement (MM). Meditative Movement is a recognized category of low-impact, low-intensity, gentle exercise comprised of four essential elements: (1) a focus on the breath, (2) body posture and/or movement, (3) a clear/calm mind, and (4) a deep state of relaxation [28]. Meditative Movement includes practices such as Tai Chi, Qigong, and various types of gentle yoga – all of which have demonstrated improved physical and psychological health outcomes across multiple populations [29,30]. As a standardized and manualized program, Tai Chi Easy is an easy-to-learn series of exercises with an emphasis on the four elements of MM. Tai Chi Easy is a form of MM combining elements of both Tai Chi and Qigong [31], was developed by a doctor of oriental medicine and founder of a national Tai Chi/Qigong instructor trainer certification program [32], and has a growing body of evidence demonstrating efficacy for improved health and well-being outcomes across populations [22,33,34].

In the current work, it is suggested that well-being predictors such as stress, mood disturbance, mindfulness, self-compassion, and body awareness may change in response to MM practices, specifically, Tai Chi Easy, and that these set of practices will, in turn, improve the associated behavioral risk factors of sleep quality and emotional eating to support positive changes in body composition. The purpose of this current single-group, pilot study was to explore relationships between well-being predictors (i.e., perceived stress, mood disturbance, mindfulness, self-compassion, body awareness) and associated behavioral risk factors (i.e., sleep quality and emotional eating) related to body composition. As a single-group exploratory pilot study, the sample size was based on the intent to explore preliminarily relationships among variables that fit with the predicted model of well-being predictors on body composition and associated behavioral risk factors, without establishing hypotheses to be tested for significance.

## Materials and Methods

### Procedures and Participants

Prior to study start, all materials, procedures, and the intervention protocol were approved by the Institutional Review Board (IRB; ID: 00005974). Study recruitment was conducted at a large Southwestern university and surrounding area businesses. Approved recruitment flyers detailed study specifics including eligibility information and study requirements. A study-specific Facebook page was created to promulgate study promotion and recruitment efforts. Interested potential participants initiated contact by calling the study phone number and/or responding via the email link on the Facebook page to set up a time to conduct eligibility screening. Notably, the current exploratory analysis was part of a larger study, with primary outcomes reported elsewhere [22].

To determine eligibility, potential participants completed a brief 5-minute phone screener with research staff. Inclusion criteria required that participants were: (1) female, between 45 and 75 years old, (2) able to participate in a low-intensity gentle movement class for 8 weeks, (3) could speak/understand English, and (4) able to attend classes on site (e.g., campus). Exclusion criteria included women who were: (1) outside of the targeted age range, (2) unable to stand up for 10 minutes, and (3) unable to walk. Eligible individuals were invited to schedule an on-site data collection appointment during which study staff reviewed the consent form with participants and once signed, engaged in data collection. Data collection (pre- and post-intervention) and intervention classes took place on campus between October 2015 and December 2017 over the course of six cohorts (ranging from 5 to 12 participants).

### Intervention

The single-group, pilot study was conducted as a pretest/posttest intervention, such that all participants received the same 8-week intervention (i.e., no control group) with once-weekly 30-minute Tai Chi Easy classes (taught by certified instructors). Tai Chi Easy intervention exercises were taught while standing up; however, participants were given the option to engage in practices while being seated and/or using a chair for balance throughout the instructed classes (with instructor guidance provided to adapt to these accommodations). Participants were encouraged to engage in daily at-home practices and received (a) a Tai Chi Easy DVD containing a demonstration video of all exercises taught in class, and (b) a hard copy log to track practice day/time.

### Measures

All study measures were collected pre- (week 0; T1) and post-intervention (week 9; T2). Pre-intervention data collection took place within the week prior to study start (week 0) and post-intervention data collection took place within the week after the last class (week 9).

The Pittsburgh Sleep Quality Index (PSQI) [35] was used to measure subjective sleep quality over the previous month. Pittsburgh Sleep Quality Index includes seven subscale components: sleep quality, sleep latency, sleep duration, habitual sleep efficiency, sleep disturbances, use of sleep medications, and daytime disturbances. Pittsburgh Sleep Quality Index contains 19 items yielding a global score ranging from 0 (high sleep quality) to 21 (low sleep quality); lower scores indicate improved levels of sleep quality. A PSQI global score of > 5 is considered indicative of significant sleep disturbance [35]. PSQI reports strong internal consistency (Cronbach’s *α* = 0.83) [35].

The subjective behavior of emotional eating was measured using the Emotional Eating (EE) subscale of the Three Factor Eating Questionnaire-18 (TFEQ-18) [36]. Items were scored on a 4-point Likert scale consisting of three subscale scores: (1) disinhibition, (2) hunger, and (3) emotional eating. Specifically, the EE subscale is comprised of three questions, with higher scores indicating greater incidence of emotional eating (i.e., indicating a maladaptive response/behavior). The EE subscale demonstrates strong internal consistency, Cronbach’s *α* = 0.87 [36].

Stress defined as state of worry or mental tension caused by a difficult situation, was measured with the Perceived Stress Scale-10 (PSS-10) [37]. Perceived Stress Scale-10 measures the degree to which respondents consider their life to be “unpredictable, uncontrollable, and overloading” over the previous month. Perceived Stress Scale-10 is scored on a 5-point Likert scale ranging from 0 = never to 4 = very often; higher scores indicate higher levels of stress. The PSS-10 has strong internal consistency (Cronbach’s *α* = 0.89) [37].

The Profile of Moods-Short Form (POMS-SF) was used to evaluate “transient, distinct mood states” in the current moment [38]. The 37-item survey uses a 5-point Likert scale ranging from 1 = not at all to 5 = extremely and is comprised of six subscales: (1) Tension-Anxiety, (2) Anger-Hostility, (3) Vigor-Activity, (4) Fatigue-Inertia, (5) Depression-Dejection, and (6) Confusion-Bewilderment; subscale scores yield strong internal consistency (Cronbach’s *α* = 0.76–0.91). POMS-SF yields seven scores – six independent subscale scores and one global scale measuring “total mood disturbance” (Cronbach’s *α* = 0.87) [38].

The subjective experience of mindfulness, or non-judgmental present moment awareness, was measured with the Cognitive and Affective Mindfulness Scale-Revised (CAMS-R) [39]. Cognitive and Affective Mindfulness Scale-Revised is comprised of 12 items and measures four specific mindfulness components experienced on a daily basis: (1) attention, (2) present focus, (3) awareness, and (4) acceptance/non-judgement. Although CAMS-R has distinctly measurable components, it yields only one total (mindfulness) score. CAMS-R-10 is scored on a 4-point Likert scale ranging from 1 = rarely/not at all to 4 = almost always; higher scores indicate higher levels of mindfulness. The scale demonstrates high internal consistency (Cronbach’s *α* = 0.78) [39].

Self-compassion was measured using the Self-Compassion Scale (SCS) [40]. The full 26-item SCS (used for the current study) includes six subscales: (1) self-kindness, (2) common humanity, (3) mindfulness, (4) self-judgment, (5) isolation, and (6) over-identification. Self-Compassion Scale yields six subscale scores and one overall total self-compassion score. The scale uses a 5-point Likert scale, 0 = almost never to 5 = almost always; higher total self-compassion scores indicate higher self-compassion. SCS subscales demonstrate strong internal consistency (Cronbach’s *α* = 0.75–0.81) as does the scale as one total measure (Cronbach’s *α* = 0.92) [40].

To measure participant’s attentiveness to bodily processes, the Body Awareness Questionnaire (BAQ) was used [41]; BAQ is comprised of 18 items and uses a 7-point Likert scale, 1 = not at all true of me to 7 = very true of me. BAQ is constructed based on the following four components: (1) Note Response or Changes in Body Process, (2) Predict Body Reaction, (3) Sleep-Wake Cycle, (4) Onset of Illness, and yields one global score. BAQ has strong internal consistency (Cronbach’s *α* = 0.82) [41].

With respect to the selected surveys, and included constructs, although not mutually exclusive, and therefore with inherent element(s) of potential overlap, scale selection was determined appropriate for our outcomes of interest. Multiple scales – PSS-10, POMS-SF, CAMS-R, SCS, BAQ – provided a more robust approach to capture potential nuances and variations within our given population. The selected scales were chosen based on the specific information needed to aptly address and answer the research questions and effectively measure the multiple constructs and outcomes of interest.

### Data Analysis

All statistical analyses were conducted using IBM Statistical Package for the Social Sciences (SPSS)-24. Demographic variables (e.g., age, race, ethnicity, education), were described using mean and standard deviation or frequency and proportion, as appropriate. Data were cleaned and the distribution of continuous variables was determined to be normal. Change scores were computed (pre-intervention subtracted from post-intervention) and used for the analyses. Correlations between the primary predictors (i.e., sleep quality and emotional eating) and well-being factors (i.e., stress, mood state, mindfulness, self-compassion, body awareness) were quantified using Pearson’s correlation coefficients; linear regression models were run to explore relationships between the dependent and independent variables.

### Correlation Coefficients

Variables entered into the correlation analysis were selected based on evidence drawn from previous literature demonstrating statistically significant relationships. Pearson’s product-moment correlations between primary outcomes and putative predictors were calculated. Correlations were considered meaningfully related when the *p*-values were equal to or less than *p* = 0.20 and were therefore considered appropriate to further examine associations. In the context of an exploratory analysis, this less conservative approach was selected to provide increased visibility of potentially significant, or of interest, variables that were potentially correlated. The strength, or effect size, of the correlations were interpreted using the following cutoff values: *r* ≥ 0.1–0.3 = small; *r* ≥ 0.3–0.5 = medium; *r* = 0.5–1.0 = large [42].

### Regression Analyses

To explore relationships between the dependent variables and independent variables, and further, to explore potential predictive value of the independent variables on the changes in dependent variable scores (post-intervention), multivariate linear regression models were fitted. Notably, all variables were continuous. Specifically, using the backward linear regression method in SPSS, regression analyses were run entering all putative predictor variables that had correlations with *p*-values of 0.20 or less. The algorithm then tested all entered variables, removing the one that made the least contribution to the model until the level of statistical significance for predictors in the final model was set at *p* = ≤ 0.05. The backward regression was done only using the putative predictor variables with Pearson correlation *p*-values of ≤ = 0.20; to these final models for each dependent outcome variable, age was entered to adjust for this variable. To evaluate the model Adjusted R2 was used. To further understand the contributions of the predictor variables, we examined the standardized coefficients (Beta), which convert the different variables to the same scale [43].

## Results

### Study Population

At baseline, 51 women were enrolled for participation; however, over the course of the intervention, 15 participants dropped out or did not complete post-intervention data, yielding *N* = 36 for final analysis. Most participants (*M* age = 53.7) were White (80.4%) and had attended ≥ 4 years of college (70.6%). The survey score means for primary and exploratory variables for pre, post, and change in score, as well as range of scores are presented in Table 1. The average number of weeks that participants attended the once-weekly class over the 8-week study duration was 6 weeks, or 6 classes total.

**Table 1.** Survey score pre/post mean, standard deviations, and changes; primary and exploratory variables

Variable	Pre Mean, SD *N* = 36	Post Mean, SD *N* = 36	Change
Primary:			
Sleep Quality	6.38, 3.22	4.56, 2.86	−0.88
Emotional Eating	2.02, 0.76	1.88, 0.69	−0.16
Exploratory:			
Perceived Stress	15.00, 7.37	12.49, 6.64	−2.36
Total Mood Disturbance	5.22, 3.42	4.74, 2.81	−0.27
Mindfulness	27.92, 5.84	29.22, 5.60	1.16
Self-Compassion	3.34, 0.59	3.54, 0.61	0.18
Body Awareness	4.54, 1.14	4.84, 1.15	0.36

### Sleep Quality

The correlations between putative predictors and dependent/outcome variables are displayed in Table 2.

**Table 2.** Pearson’s correlation coefficients of primary and exploratory variables

Variable		1	2	3	4	5	6	7
Primary:								
	1. Sleep Quality	–	0.03	**0.46**	**0.45**	−0.03	**−0.03**	**−0.43**
	2. Emotional Eating	0.03	–	**0.37**	**0.30**	**−0.41**	**−0.66**	**−0.29**
Exploratory:								
	3. Perceived Stress	**0.46**	**0.37**	–	**0.60**	**−0.23**	**−0.38**	**−0.48**
	4. Mood State	**0.45**	**0.30**	**0.60**	–	**−0.32**	**−0.24**	**−0.30**
	5. Mindfulness	−0.30	**−0.41**	**−0.23**	**−0.31**	–	**0.30**	**0.35**
	6. Self-Compassion	**−0.30**	**−0.66**	**−0.38**	**−0.24**	**0.30**	–	**0.46**
	7. Body Awareness	−0.43	−0.29	−0.48	−0.38	0.35	0.46	–

Bolded values significant at ≤ 0.2.

Sleep quality scores (as assessed by PSQI; higher scores indicating poor sleep quality) decreased from baseline (*M* = 6.38, SD = 3.22) after intervention (*M* = 4.56, SD = 2.86). Sleep quality showed a weak and positive correlation with perceived stress, *r* = 0.46, *p* = 0.03. Sleep quality and mood state (total mood disturbance with higher scores indicating higher disturbance of mood) also demonstrated a weak and positive correlation, *r* = 0.45, *p* = 0.03. Sleep quality was weakly and negatively correlated with self-compassion, *r* = −0.30, *p* = 0.18. Lastly, sleep quality showed a weak and negative correlation with body awareness, *r* = −0.43, *p* = 0.05.

Emotional eating scores (as assessed by the EE subscale of the Three Factor Eating Questionnaire-18 clinical interpretation, with higher scores indicating greater instance of emotional eating) decreased from baseline (*M* = 2.02, SD = 0.76) following intervention (*M* = 1.88, SD = 0.69). There was a weak and positive correlation between emotional eating and perceived stress, *r* = 0.37, *p* = 0.03. Emotional eating and mood state (i.e., total mood disturbance) showed a weak and positive correlation, *r* = 0.30, *p* = 0.07. Emotional eating and mindfulness were weakly and negatively correlated, *r* = −0.41, *p* = 0.01. There was a moderate and negative correlation between self-compassion and emotional eating, *r* = −0.66, *p* = < 0.01. Finally, results showed a weak and negative correlation between body awareness and emotional eating, *r* = −0.29, *p* = 0.10.

### Regression Models

With respect to the regression models fitted in this study (Table 3), the issue of multicollinearity (highly correlated independent variables, *r* > 0.7) [43] was not relevant; therefore, was not considered a threat to the regression models, therefore none of the independent variables had to be eliminated. Additionally, there were no observed outliers among the psychoemotional variables, and as such, no data were removed.

**Table 3.** Summary of linear regression analyses for variables predicting sleep quality and emotional eating

Variable	Raw B	95 % CI B		Standardized	
		Lower	Upper	B	*p*
Sleep Quality					
Age	0.019	−0.097	0.135	0.458	0.738
Change in Perceived Stress	0.152	0.015	0.290	0.067	0.032
Emotional Eating					
Age	0.006	−0.015	0.026	0.079	0.569
Change in Self-Compassion	−0.615	−0.867	−0.363	−0.682	0.000

Backward linear regression analyses resulted in only one independent variable – perceived stress – which approached a statistically significant predictor of sleep quality, *F* (2, 20) = 2.71, *p* = 0.09. Although stress did not meet the generally accepted *p* = ≤ 0.05 threshold after backward stepwise regression with stated predictors; however, when adjusted for age, perceived stress was a significant predictor of sleep quality, Beta = 0.46, 95% CI [0.02, 0.29], *p* = 0.03 with an adjusted R2 value suggesting stress and age explained 13.4% of the variance in change in sleep quality. With respect to emotional eating, only self-compassion was a significant predictor, *F* (2, 31) = 12.54, *p* < .01.

To examine the predictive potential of factors on emotional eating, backward linear regression analyses showed only one independent variable, self-compassion, as a statistically significant predictor of the variance (*F* (2, 31) = 12.54, Beta = −0.68, 95% CI [−0.87, −0.36], *p* = < .01). The final model included self-compassion and age with the adjusted R2 value indicating that 42.1% of the variance in emotional eating was explained by self- compassion and age.

## Discussion

The purpose of this single-group pilot study was to explore relationships between select well-being factors (i.e., stress, mood state, mindfulness, self-compassion, body awareness) and the associated behavioral risk factors of sleep quality and emotional eating on changes in body composition in midlife and older women. Further, the current study aimed to determine if these independent variables of interest were significant predictors of the sleep quality and/or emotional eating outcomes, potentially explaining variance in the changed pre- to post-intervention scores. Findings for correlations and regression models partially support the study hypotheses (See Tables 2 and 3).

Sleep quality as measured by the PSQI was found to improve over the course of intervention and is related to multiple facets of well-being. Overall group sleep quality was indicative of significant sleep disturbance at baseline and revealed improvement following intervention. Post-intervention assessment revealed improved sleep quality as the group mean PSQI score fell below the cutoff indicative of significant sleep disturbance. Quality of sleep demonstrated a weak and positive relationship with both perceived stress, which relates to psychological well-being and mood disturbance, which, in turn, relates to emotional well-being. The expected direction of these correlations is well supported by previous research [44]. Additionally, sleep quality was negatively correlated with self-compassion, an additional construct tied to psychological well-being. The relationship between sleep quality and self-compassion is becoming more well-recognized in literature [21]. As the sleep quality score decreased (indicating an improvement), self-compassion increased demonstrating a relationship between physical and psychological facets of well-being. Although less studied, sleep quality showed a negative correlation with body awareness (i.e., decreased sleep scores representing improved sleep were related to increased body awareness).

Reported instances of emotional eating decreased from baseline following intervention. Emotional eating had a positive correlation with perceived stress and mood state which is strongly supported by previous research across populations [45,46]. Emotional eating and mindfulness were negatively correlated such that with increased mindfulness there was decreased emotional eating – findings that are also aligned with previous results [47]. Further, emotional eating showed a negative correlation with self-compassion – as self-compassion increased, emotional eating decreased which demonstrated a relationship between emotional and psychological well-being. These findings align with work related to eating behaviors in the context of self-compassion interventions [48]. Finally, there was a negative association between emotional eating and body awareness, such that higher body awareness was correlated with decreased emotional eating. The concept of body awareness involves “…attentional focus on and awareness of internal body sensations” [49] (p1) and is imperative to understand in the context of emotional eating. Body awareness can be viewed as either adaptive or maladaptive. In the adaptive domain, body awareness heightens sensitivity to what one’s internal experience is and recognizes bodily needs. The maladaptive domain of body awareness is demonstrated when an individual hyper-focuses on a “negative” (bodily) sensation or aspect of physical well-being, which tends to amplify and exacerbate these negative feelings [49]. In the framework of a mind-body intervention, we suggest that emotional eating (eating in response to negative emotion) and body awareness (attention/awareness to body sensations) may serve to inform and support each other such that heightened awareness of each may facilitate more healthy choices – if one is aware of the emotional experience (i.e., stress) *and* their bodily sensation (i.e., aching, satiation) one may then be able to attune to what is needed. “Awareness” may be the link between emotional eating and body awareness that helps promote improved self-regulatory behaviors.

The results of the regression analysis suggested that: (1) perceived stress explained a portion of the variance in sleep quality and (2) self-compassion explained a relatively large amount of the variance in emotional eating. Other research supports these findings, as the measure of perceived stress is unquestionably related to sleep quality, such that increased stress (i.e., perception, elevated hormones) inhibits and/or disrupts healthy sleep quality [50]. Self-compassion, the attitude of being kind to and caring toward oneself, is aptly positioned to enhance self-regulatory responses which may attenuate the maladaptive behavior of emotional eating. The direct practices of self-compassion are intended to create a felt sense of kindness and caring in a way that is non-judgmental in the present moment; importantly this construct is, by definition, placed in the context of suffering, such that one brings this way of being to oneself when met with challenging and/or difficult situations. Bringing kind attention to oneself when struggling with difficult emotions (e.g., stress, mood disturbance) may attenuate the, oftentimes, habituated, and maladaptive response of emotional eating. With respect to the construct of emotional eating, self-compassion was a statistically significant predictor in the final regression model.

### Limitations

The current single-group pilot study has noted limitations. First, the results and interpretation are limited by the small sample size (*N* = 36) and homogeneity (highly educated and lacking racial/ethnic diversity). Secondly, lack of a control group limits the ability to: (1) demonstrate efficacy, (2) compare (intervention) results to that of a population who did not receive the intervention, and (3) examine mediators in the context of comparison group. The limited duration/frequency (8 weeks, 30 minutes, once weekly), with some participants attending less than the full set of (8 weeks) classes, may have compromised the ability to see additional changes in outcomes, particularly body composition (e.g., percent body fat). A longer intervention with a matched control group may have yielded more robust changes, correlations, and the potential to explore predictors as mediators driving change. Lastly, there were additional variables not explored in the correlation matrix and therefore not entered into the regression models. There are a host of variables related to the body composition and the associated behavioral risk factors of sleep quality and emotional eating – it is possible that physical activity, dietary intake, use of medications, and prior medical diagnosis may have factored into the regression models and explained additional variance in the dependent variables.

## Conclusion

While considerable intervention-driven research in the fields of exercise and diet has demonstrated improved body composition and reduced obesity, long-term sustained results are limited. Findings from the current pilot study suggest that, in the context of a MM intervention, well-being predictors (i.e., stress, mood state, mindfulness, self-compassion, and body awareness) are correlated with associated behavioral risk factors of sleep quality and emotional eating, both strongly related to body composition. Further, results demonstrated that perceived stress and self-compassion may act as significant predictors on the associated behavioral risk factors of sleep quality and emotional eating. Additional research is needed to continue to explore and explain the relationships between select well-being predictors and associated behavioral risk factors targeted toward improved body composition among midlife and older women.
